# Revealing the paradox of drug reward in human evolution

**DOI:** 10.1098/rspb.2007.1673

**Published:** 2008-03-19

**Authors:** Roger J Sullivan, Edward H Hagen, Peter Hammerstein

**Affiliations:** 1 Department of Anthropology, California State UniversitySacramento, CA 95819, USA; 2 Department of Psychiatry and Behavioral Sciences, UC Davis School of MedicineSacramento, CA 95817, USA; 3 Department of Anthropology, Washington State UniversityVancouver, WA 98686-9600, USA; 4 Institute for Theoretical Biology, Humboldt University10115 Berlin, Germany

**Keywords:** reward, cytochrome P450, neurotoxins

## Abstract

Neurobiological models of drug abuse propose that drug use is initiated and maintained by rewarding feedback mechanisms. However, the most commonly used drugs are plant neurotoxins that evolved to punish, not reward, consumption by animal herbivores. Reward models therefore implicitly assume an evolutionary *mismatch* between recent drug-profligate environments and a relatively drug-free past in which a reward centre, incidentally vulnerable to neurotoxins, could evolve. By contrast, emerging insights from plant evolutionary ecology and the genetics of hepatic enzymes, particularly cytochrome P450, indicate that animal and hominid taxa have been exposed to plant toxins throughout their evolution. Specifically, evidence of conserved function, stabilizing selection, and population-specific selection of human cytochrome P450 genes indicate recent evolutionary exposure to plant toxins, including those that affect animal nervous systems. Thus, the human propensity to seek out and consume plant neurotoxins is a paradox with far-reaching implications for current drug-reward theory. We sketch some potential resolutions of the paradox, including the possibility that humans may have evolved to counter-exploit plant neurotoxins. Resolving the paradox of drug reward will require a synthesis of ecological and neurobiological perspectives of drug seeking and use.

## 1. Introduction

The use of psychoactive substances is one of the most perplexing human behaviours. Some substances cause immeasurable harm to individuals and societies (e.g. heroin) or impose a tremendous social burden in the form of preventable chronic illnesses (e.g. tobacco), while others appear to be mostly harmless and are widely enjoyed by people around the world (e.g. coffee and chocolate). Historically, a broad range of psychosocial, behavioural and neurobiological theories seeking to understand drug phenomena are unified by the notions of reward and reinforcement (Thorndike 1911). According to these theories, recreational drugs reward and/or reinforce consumption, often via hedonic effects (Wise & Rompre 1989; Everitt & Robbins 2005; Kalivas 2005; Koob & Le Moal 2005; Nestler 2005).

Most commonly used psychoactive drugs are plant secondary metabolites (e.g. alkaloids) or their close chemical analogues (table 1, see also further detail in table 1, electronic supplementary material). Evolutionary biologists studying plant–herbivore interactions have convincingly argued that many plant secondary metabolites, including alkaloids such as nicotine, morphine and cocaine, are potent neurotoxins that evolved to deter consumption by herbivores (Karban & Baldwin 1997; Roberts & Wink 1998).^1^ On the other hand, neurobiology's reward model sees interactions between drugs and the nervous system as rewarding and reinforcing. Hence, in their current forms, neurobiology's reward model and evolutionary biology's punishment model appear to be incompatible. We term this incompatibility the *paradox of drug reward*.

Several theorists have attempted to explain drug reward from an evolutionary perspective emphasizing fitness consequences (Tooby & Cosmides 1990; Nesse & Berridge 1997; Johns 1999; Smith 1999; Kelley & Berridge 2002; Newlin 2002). In this view, behaviours beneficial to an animal's reproductive success are rewarded and/or reinforced by positive emotions, while behaviours with fitness-impairing consequences are discouraged with negative emotions. This perspective holds that drugs of abuse subvert natural reward circuits by creating a signal in the brain falsely indicating the arrival of a huge fitness benefit (positive reinforcement), and by blocking painful feelings or affect states, ‘short circuiting’ the adaptive functions of negative emotions (Nesse & Berridge 1997).

This current evolutionary interpretation of brain function and reward could potentially resolve the paradox, but carries with it several assumptions with which we intend to take issue. These are summarized below.

- The widespread availability of drugs in the present is an evolutionary *novelty*, or *mismatch*; meaning that the brain, and its putative reward centres, evolved in ancestral drug-free environments (Tooby & Cosmides 1990; Nesse & Berridge 1997; Smith 1999). To the contrary, we will argue that the evolutionary biological evidence strongly indicates that humans and other animals have been exposed to drugs throughout their evolution, and will base this argument on an analysis of the evolution of hepatic enzymes (cytochrome P450) that metabolize environmental chemicals.
- The brain and its reward centres are *inherently vulnerable* to drugs (Nesse & Berridge 1997). This idea follows from the ‘mismatch’ assumption. If drug exposure is an evolutionary novelty, then it logically follows that the brain does not have evolved defences to protect it from psychoactive substances. If such exposures were not novel then, as (Nesse & Berridge 1997) point out, ‘Hundreds of generations of exposure would likely shape resistance to [drugs'] allure and …deleterious effects.’ We will use evidence from the genetics of drug metabolism to show that humans have been exposed to plant neurotoxins throughout their evolution, and to indicate that we are unlikely to be *inherently vulnerable* to drugs.
- Drugs of abuse are intrinsically *rewarding* and that sentient rewards correlate with *fitness consequences*. To the contrary, we will argue that commonly used drugs are often experienced as affectively unpleasant by neophyte users (i.e. non-rewarding) and are identified physiologically as toxic (non-rewarding) in the nervous system.

Among the points of contention described above, the key question is that of ‘novelty’ or mismatch—whether or not our ancestors were exposed to psychoactive neurotoxins. Our rejection of the assumption that drugs are evolutionarily novel, we will argue, leaves the paradox of drug reward unresolved. Although we see no obvious resolution to the paradox, we conclude by sketching some possibilities, including a consideration of the potential adaptive outcomes of an evolutionary exposure to plant neurotoxins in humans.

## 2. Acute drug reward: the neurobiological perspective

Neurobiological theory of drug use distinguishes between initial seeking and use, and the longer term phenomena of drug tolerance and addiction. Causal theories of both ‘stages’ of drug use employ the concepts of reward or reinforcement: as motivation for drug seeking and in the acute effects of initial use, and in the maintenance of long-term drug use and the neuroplastic changes that occur in response to chronic drug exposure. It is not surprising then, that the *motivations* for neophyte drug use are often conflated by a failure to distinguish between the causes of initial and long-term drug use (Wallace 2004). Our focus is on the most enigmatic phase of drug use—initial drug seeking and its acute effects, which we detail here, and not on the distinct processes of dependence and addiction (for recent reviews of the neurobiology of addiction see Kalivas & Volkow (2005) and Nestler (2005); for a list of theories of addiction, see West (2001)). Note that neither stage of drug-use theory typically incorporates evolutionary insights (cf. Lende 2007).

The leading neurobiological model of acute drug reward and reinforcement is the mesolimbic dopamine system (MDS). In simplified terms, the MDS comprises dopaminergic neurons projecting from the ventral tegmental area (VTA) in the midbrain to the nucleus accumbens (NAc) in the basal forebrain. The degree of activity of the dopaminergic projections affects concentrations of extracellular dopamine in the NAc (Kelley & Berridge 2002; Koob & Le Moal 2005; Nestler 2005).

An influential interpretation of dopamine function in the MDS followed from the observation that dopamine receptor blockade induced an ‘anhedonia’ in rats, which was not explained by sedation or motor side effects (Wise *et al*. 1978). In other words, under what is sometimes referred to as the *hedonia* hypothesis, dopamine mediates the unconditioned pleasure produced by food, sex, etc., as well as the conditioned pleasure produced by secondary reinforcers like drugs.

The discovery that dopamine neurons fail to respond when animals receive an *anticipated* reward contradicts the hedonia hypothesis, suggesting instead that the response of midbrain dopaminergic neurons might encode prediction errors—the difference between predicted and obtained rewards—rather than absolute reward. Under this hypothesis, unpredicted rewards elicit activation of midbrain dopaminergic neurons (positive prediction error), fully predicted rewards elicit no response, and the omission of predicted rewards induces a depression (negative prediction; Schultz 1998).

The *incentive salience* hypothesis (Robinson & Berridge 1993; Berridge & Robinson 1998) represents a further revision of the original hedonia hypothesis. This perspective stresses that manipulation of dopamine transmission has a powerful impact on motivation without changing hedonic reactions, and that ‘wanting’ is neurologically, psychologically and conceptually distinct from ‘liking’. In this view, the MDS mediates wanting and not liking (i.e. not hedonia).

Despite diverse mechanisms of action and heterogeneous effects in the brain and periphery, initial and acute exposure to all drugs of abuse is thought to interfere with the normal functions of the MDS by increasing dopaminergic transmission in the NAc (Nestler 2005). For example, stimulants such as amphetamines and cocaine directly increase dopaminergic transmission in the NAc; opiates inhibit GABAergic interneurons in the VTA by disinhibiting VTA dopamine neurons projecting to the NAc; and nicotine seems to activate VTA dopamine neurons both directly and indirectly via stimulation of glutamatergic terminals innervating dopamine cells (Nestler 2005; Lüscher & Ungless 2006). Although the precise effects of increased dopamine levels are not yet resolved, this drug-induced increase is widely believed to reward and/or reinforce drug use (Kelley & Berridge 2002; Koob & Le Moal 2005; Nestler 2005).

## 3. Plant chemical punishment of herbivores: the evolutionary biological perspective

The relationship between living plants and the animals that feed on them is antagonistic, forged by an intense and ongoing evolutionary arms race. In the evolution of life, autotrophs—plants, algae and some bacteria that synthesize organic compounds from inorganic sources of carbon and energy—became the ultimate source of raw materials and energy for heterotrophs—the animals, fungi, protists, bacteria and viruses that constitute the Earth's remaining biota.^2^

Plants and other autotrophs have evolved an impressive array of chemical defences to deter heterotrophic predators (insect and vertebrate herbivores, fungi and microbes; Wink 1998). All cellular organisms possess internal signalling systems to regulate metabolism, growth and reproduction, which depend on small quantities of simple molecules such as amines, peptides, steroids and lipids. Although such systems are efficient, they are vulnerable to manipulation by adversaries. As a key facet of their chemical defence, many autotrophs have coevolved compounds that are either identical to or closely mimic these signalling molecules; thus enabling autotrophs to subject herbivores to disrupting chemical attacks. For example, one or more plant alkaloids have been identified that interfere with nearly every step in neural signalling. Targets include neurotransmitter synthesis, storage, release, binding, deactivation and reuptake, ion channel activation and function, and key enzymes involved in signal transduction (Wink 2000).

Paradoxically, the same properties invoked to explain why common drugs like caffeine, nicotine and cocaine are toxic are also those invoked to explain why these compounds are rewarding. It is therefore important to stress that these and other addictive drugs appear to have evolved only because they successfully *deterred*, not rewarded or reinforced, plant consumption. Among drugs of abuse, the data are particularly clear for nicotine, an alkaloid that binds to acetylcholine receptors. *Nicotiana attenuata* is an important model species for the analysis of plant–herbivore interactions involving nicotine. It is a domesticated North American tobacco plant that is attacked by over 20 different herbivores, ranging from mammalian browsers to intracellular-feeding insects (Baldwin 2001). These attacks elicit a battery of defensive responses, including nicotine production.

For example, under natural conditions, *Nicotiana* with transgenically downregulated nicotine production lost threefold more leaf area to herbivores than did wild-type plants, supporting a defensive function for nicotine (Steppuhn *et al*. 2004). *Nicotiana* treated with jasmonic acid (JA) to boost concentrations of nicotine and other defences had less leaf loss to mammalian browsers, lower mortality rate and produced more viable seed than size-matched controls. The insect herbivore, tobacco hornworm (*Manduca sexta*) showed dramatically lower survivorship and growth on JA-treated plants than on untreated controls. However, for plants not subject to herbivory, JA treatment reduced seed production by 26% relative to controls, demonstrating that chemical defences are expensive to produce. *Nicotiana* has therefore evolved to allocate chemical defences strategically by concentrating them in the most valuable parts of the plant, such as young leaves, stems and reproductive organs, and by modulating its production according to the type of herbivore and extent of leaf damage (Baldwin 2001).

Herbivores have coevolved a number of countermeasures in response to the evolution of autotroph chemical defences including: chemosensors that permit selective feeding on less toxic tissues, compounds that prevent or attenuate induction of autotroph chemical defences, mechanisms that extract benefits from defensive chemicals (e.g. by metabolizing them or sequestering them to aid heterotrophic chemical defence against predators), symbiotic relationships with microbes to detoxify or extract nutrients from plant defences, cellular membranes for multidrug transport and, perhaps most commonly, enzymes that detoxify plant secondary compounds (Karban & Agrawal 2002). We will return to the latter topic shortly.

## 4. Is drug exposure an evolutionary novelty?

Neurobiologists have made a strong case that drug seeking and use is intimately related to neural circuitry involved in reward and/or reinforcement. However, evolutionary biologists have made an equally strong case that plant neurotoxins evolved to *punish*, not reward, plant consumers, and that it is in the fitness interests of both plant and consumer that the consumer is averse to the plant's defensive toxins. From the perspective of evolutionary ecology, plants should not have evolved defensive chemicals that easily trigger reward in consumers, and consumers should not have evolved neural circuitry that readily but inadvertently rewards or reinforces consumption of numerous neurotoxins.

Several evolutionary theorists have argued that the MDS evolved to reinforce behaviours that successfully resulted in food acquisition, mating and other fitness-enhancing outcomes, and is maladaptively triggered by *evolutionarily novel* exposure to psychoactive plant toxins (Tooby & Cosmides 1990; Nesse & Berridge 1997; Johns 1999; Smith 1999; Kelley & Berridge 2002; Newlin 2002). If exposure to plant neurotoxins is evolutionarily novel, then humans should exhibit little evidence of evolved countermeasures.

### (a) Detoxification enzymes: a coevolved countermeasure in humans and other animals

Here we explore a key superfamily of detoxification enzymes to assess whether they support the hypothesis that exposure to plant neurotoxins is evolutionarily novel for humans. The principal heterotroph detoxification enzymes are the cytochrome P450 (CYP) haemoproteins. CYPs are ubiquitous in Bacteria and Eukarya, and have been found in many Archaea species, suggesting that the ancestral CYP gene evolved approximately 3.5 Gyr ago. The metabolism of endogenous fatty acids and steroidogenesis appear to have been the original (and still central) functions of most CYP genes. With the rise of terrestrial plants and animals approximately 400 Myr ago, the major functions of CYPs expanded to encompass the detoxification of dietary phytochemicals via a coevolutionary process involving dozens of gene duplication events (Nelson 1999; Lewis 2001). There are approximately 76 CYP families known in animals, with 57 of these present in humans (Nelson *et al*. 2004).

Although CYPs are found in many tissues, in humans and other mammals they are concentrated in the liver, where they catalyse the oxidation of a wide range of endogenous and exogenous chemicals in phase I metabolism. CYP oxidases introduce an atom of molecular oxygen into the structure of a lipophilic substrate (such as a toxin/drug) to render it more hydrophilic prior to conjugation to a carrier molecule in phase II metabolism for export from the body. In mammals, CYPs are responsible for oxidizing over 90% of drugs and other xenobiotics (Lewis 2001).

Several CYP families are highly conserved across species, whereas others are quite variable. The conserved CYP 5 and higher families have endogenous functions such as bile acid metabolism and cholesterol and steroid biosynthesis, and show remarkable cross-species similarity. For example, 21 out of the 22 human and mouse genes in these families are orthologous (Nelson 1999).

By contrast, most of the drug-metabolizing enzymes are in the variable group (Nelson 1999). The CYP 2 and CYP 3 families, for example, are phylogenetically divergent with 16 human CYP 2 genes and 4 CYP 3 genes, as opposed to 51 CYP 2 and 8 CYP 3 genes in mice (Nelson *et al*. 2004). There is only one 2D gene (2D6) in humans, whereas there are nine 2D genes in mice (Nelson *et al*. 2004). A comparison of the human genome with the initial sequence of the chimpanzee genome similarly found rapid evolutionary divergence in xenobiotic-metabolizing genes (The Chimpanzee Sequencing and Analysis Consortium 2005), as well as divergence in genes expressed in the liver (Khaitovich *et al*. 2005). The latter finding is supported by *in vivo* pharmacokinetic studies. For instance, systemic clearance of propranolol, verapamil, theophylline and 12 other synthetic drugs in chimpanzees and humans ranged from close to parity to a 10-fold variation, with CYP2D enzyme activity approximately 10 times higher in the chimpanzee, a species that notably subsists primarily on plants (Wong 2004).

The emergence of xenobiotic-metabolizing CYP in animals at about the same time as the evolution of terrestrial plants, the localization of cross-species variation in CYP genes within the xenobiotic-metabolizing subset, and the large species differences in drug metabolism suggest species-specific adaptation to frequently encountered plant toxins and other environmental chemicals.

### (b) Evolution in human xenobiotic-metabolizing cytochrome P450

The mammalian cytochrome P450 phylogenetic data are compelling evidence of a long evolutionary history of exposure to plant toxins. As mammals, humans have phylogenetically ‘inherited’ the cytochrome P450 system for detoxification of environmental chemicals. This fact alone would seem to falsify the hypothesis that human exposure to drugs is evolutionarily novel—that there has been a mismatch between contemporary drug-profligate and ancestral environments that were ‘drug’ free. But humans are taxonomically unique in several respects, particularly in regard to the relative magnitude of culture and technology. A scenario that might preserve the mismatch hypothesis is that the evolving human nervous system was ‘protected’ in a unique cultural or ecological niche that excluded plant neurotoxins. In this scenario, the phylogenetically inherited mammalian P450 adaptation was superfluous until the proliferation of drugs in the modern era. The *Homo* genus with its meat-rich diet and detoxification technology (e.g. fire), probably had reduced toxins in its diet relative to the chimpanzee (Ingelman-Sundberg 2005). However, several lines of evidence indicate that our ancestors were regularly exposed to plant neurotoxins, and do not constitute a special case in mammalian evolution.

#### (i) Conserved function

Perhaps the most compelling argument that humans have experienced relatively recent selection pressures from plant toxins is that xenobiotic-metabolizing function is conserved. Psychoactive plant toxins are substrates of CYP enzymes (table 2) with enzyme activity levels similar to those for endogenous hormones and essential fatty acids (table 2 in the electronic supplementary material). If plant toxins were *not* a recent selection pressure on humans, then loss of enzyme function through genetic drift would be expected. For the majority of phenotypes, loss of function from drift does not appear to have occurred.

#### (ii) Stabilizing selection

Solus *et al*. (2004) sequenced 11 out of the 23 genes in the xenobiotic-metabolizing CYP1, CYP2 and CYP3 families. DNA was sampled from 93 ethnically diverse humans, including Caucasians, African-Americans and Asians. Although measures of genetic diversity indicated these genes were comparatively rich in variation (mostly as a consequence of low-frequency polymorphism), four independent measures also indicated that these genes are under selection against non-synonymous amino acid changes in coding regions. Across all genes, for example, the ratio of variation in non-synonymous versus synonymous coding regions, *π*_*NS*_/*π*_*S*_, was 0.27, signifying stabilizing selection and thus a recent evolutionary history of exposure to xenobiotics such as plant toxins. For several key enzymes, CYP1A2, CYP2A6, CYP2C19 and CYP2E1, these ratios were particularly low (*π*_*NS*_/*π*_*S*_=0.08, 0.08, 0.06 and 0.08, respectively).

#### (iii) Population-specific selection

Most polymorphisms in CYP genes are of low frequency, but several are found with relatively high frequency in certain populations. In some cases, high-frequency polymorphisms can be plausibly associated with the local plant ecology. Ethiopia, Saudi Arabia and Turkey, for example, have very high frequencies of 2D6 ultrametabolizers: individuals with multiple functional copies of 2D6 genes (Aynacioglu *et al*. 1999). This pattern suggests positive selection for CYP2D6 (Ingelman-Sundberg 2005), an enzyme that metabolizes opiates and amphetamine-like compounds, together with other substrates. Perhaps not coincidentally, the opium poppy is native to the Turkish region, and *khat* (a plant containing amphetamine-like compounds) is native to North East Africa and is ubiquitously chewed in Ethiopia and the Arabian Peninsula (Sullivan & Hagen 2002; Sullivan 2003).

#### (iv) Reduced selection

In most cases, no compelling explanation exists for high-frequency population-specific CYP polymorphisms. Some high-frequency polymorphisms produce low or non-functioning enzymes that suggest reduced or no selection by certain categories of xenobiotics. Such evidence could undermine the argument that humans have been under recent selection by plant toxins; we address this potential objection by investigating two CYP genes in detail. CYP2D6 is well studied and constitutes approximately 19% of total drug metabolism *in vivo* (Lewis 2001), with more than 100 alleles identified (Nelson *et al*. 2008). CYP2A6 is less researched, but is important here as the principal metabolizing enzyme of nicotine. CYP2A6 constitutes 2–3% of total drug metabolism in humans (Lewis 2001) and has more than 30 currently recognized alleles (Nelson *et al*. 2008). Together, CYP2D6 and CYP2A6 are representatives of current descriptive knowledge in human pharmacogenetics. The majority of 2D6 and 2A6 alleles have low frequencies, consistent with stabilizing selection, but a few have high frequencies in some populations. Table 3 lists several alleles for which *in vivo* enzyme activity has been measured (see also table 3 in the electronic supplementary material).

Some of the strongest evidence of reduced selection for, or selection against, a particular CYP enzyme occurs in Asian populations, among which the highest frequencies of non-functioning CYP2A6 alleles are observed. Assuming Hardy–Weinberg equilibrium, the allele frequencies in table 3 indicate that approximately 4% of Japanese are non-metabolizers (^*^4/^*^4), and another 16% lack a normal metabolizing allele (e.g. ^*^7/^*^7, ^*^9/^*^9, ^*^4/^*^7, ^*^4/^*^9). Nonetheless, individuals with the latter genotypes still metabolize nicotine adequately, albeit at somewhat reduced levels. In one study of Koreans, for example, the cotinine/nicotine ratio (an *in vivo* measure of enzyme activity) was 10.4 for the ^*^1A/^*^1A genotype, 7.7 for ^*^1A/^*^9 and 4.3 for ^*^9/^*^9 (Yoshida *et al*. 2003). Similarly, up to 10% of some Caucasian populations and 19% of South African San are poor CYP2D6 metabolizers (Sommers *et al*. 1988; Bernard *et al*. 2006).

Although humans have probably been exposed to fewer plant toxins than our non-human ancestors (Ingelman-Sundberg 2005), we believe that these population-level patterns of 2A6 and 2D6 enzyme activity more strongly support a hypothesis of relatively recent population-specific selection by particular categories of xenobiotics, rather than a global near absence of exposure to plant toxins across human evolution (Sullivan 2003). For example, although Asians have high frequencies of non-functional 2A6 alleles, they also have low frequencies of non-functional 2D6 alleles, a pattern that is more or less reversed in Caucasians (table 3). If Asians and Caucasians had not been subjected to selection from xenobiotics, then both populations should have high frequencies of non-functional alleles for both genes. Overall, 2D6 and 2A6 adequately metabolize typical substrates in 90% of individuals, albeit at variable rates.

We interpret the conserved function in human CYP enzymes, the statistical evidence of stabilizing selection and the existence of both species- and population-specific polymorphisms as evidence that humans have undergone relatively recent selection by plant toxins frequently encountered in local environments. This hypothesis, if correct, has important implications for reward models of drug seeking and acute drug use.

## 5. The paradox of human drug use

### (a) Are we inherently vulnerable to drugs?

The notion that we are inherently vulnerable to drugs is implicit in neurobiological models of the mammalian MDS. The current assumption is that the MDS is easily triggered by a broad range of neurotoxins because it was not exposed to such toxins during its evolution (Nesse & Berridge 1997). The coevolution of the xenobiotic-metabolizing CYP families contradicts this view by demonstrating that heterotroph signalling systems have successfully endured a relentless chemical assault by autotrophs for hundreds of millions of years.

The long exposure to plant neurotoxins indicated by the CYP data make it unlikely that humans, or other mammals, are inherently vulnerable to drugs. This conclusion amounts to a rejection of the conventional evolutionary explanation of drug use, dependent as it is on the notion that the MDS evolved in the absence of selection pressures from plant neurotoxins (Nesse & Berridge 1997) or, in broader theoretical terms, that the reward and/or reinforcement functions of the MDS were somehow exempt from the implications of evolutionary biology's punishment model.

To reiterate our broader argument here, the phylogenetic evidence for coevolution of animal CYP and plant toxins reinforces the evolutionary biological perspective that plant neurotoxins evolved because they punished and deterred consumption by herbivores (Karban & Baldwin 1997; Roberts & Wink 1998), and is in direct conflict with neurobiology's reward model which sees drug use as rewarded and reinforced in the MDS. We termed this incompatibility the *paradox of drug reward*. Before sketching potential resolutions of the paradox of drug reward, we respond to several objections to our argument that emerge directly from the substantial data generated in support of current reward models like the MDS drug-reward pathway.

### (b) Does initial drug use elicit hedonic rewards and false-positive fitness signals?

Nesse & Berridge (1997) and others (Tooby & Cosmides 1990; Johns 1999; Smith 1999; Kelley & Berridge 2002; Newlin 2002) propose that positive and negative affective experiences and sensations are related to fitness consequences, and that drugs interfere with affectively mediated fitness signals. We find this perspective problematic in several ways.

First, commonly used drugs have multiple nervous system ‘targets’ and may activate physiological responses that are unpleasant or physiological systems that do not mediate affective experiences. For example, the widely used drug arecoline (betel nut) not only binds to muscarinic receptors in the brain, but also exerts potent effects in the peripheral nervous system (PNS) inducing tremor, face flushing, sweating, changes in heart rate and blood pressure, salivation, nausea and broncoconstriction (Chu 1993, 1995). The unpleasant consequences of PNS-binding sites are shared by all of the most commonly used plant drugs (e.g. tobacco, betel nut, khat, cola nut, coffee, tea, coca, cannabis). In regard to tobacco, Eissenberg & Balster (2000) demonstrated that neophyte users typically experience nausea and other aversive affects, not hedonic rewards. Most commonly used plant drugs are correctly and unsurprisingly ‘recognized’ as toxins by most new users, both physiologically and affectively, and the physical and affective responses are accurate warnings of fitness costs, rather than a false ‘positive’ signal of a fitness benefit.

**Figure 1:**
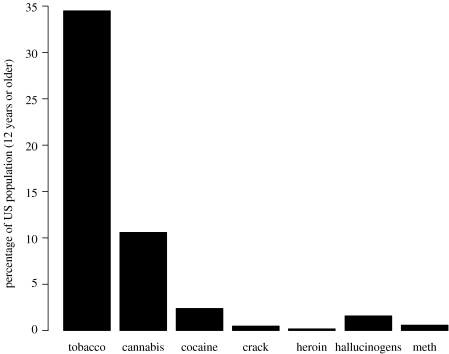
Data from the US National Survey on Drug Use and Health 2004: drug use in the last year.

Second, modern euphoric drugs, like heroin, might represent a genuine evolutionary mismatch—drugs that are vastly more pleasurable than any neurotoxins occurring naturally in ancestral environments (Nesse & Berridge 1997; Smith 1999; Nesse 2002). Yet, in population terms, euphoric drug use is trivial when compared with mundane drugs such as tobacco, cannabis and betel nut. We compiled data from the 2004 annual federal survey of drug use in the USA, to show that regular users of heroin are an extremely small proportion of the population (0.2%) and even the numbers of regular users of cocaine, ‘crack’ and amphetamines are markedly smaller (2.4, 0.5 and 0.6% of the population, respectively) than the proportions of tobacco and cannabis users (34 and 11%, respectively, ‘used in the last year’; figure 1). These data suggest that the exception—use of euphoric drugs by very small proportions of human populations—has been used to prove the ‘rule’ of hedonic drug reward. Stiff legal penalties might partially explain the exceptionally low frequency of euphoric drug consumption (DuPont & Voth 1995), but they obviously cannot explain the high frequency of non-euphoric drug consumption. Euphoric drug use is a poor model for a general theory of human, or mammalian, drug use. We suggest that commonly used non-euphoric drugs should be the basis for new models of human substance use that reflect major, rather than minor, population-use trends.

### (c) Potency

Smith (1999) and Nesse (2002) have argued that even commonly used drugs are more potent and are more likely to be encountered today than they were by our ancestors. We have countered elsewhere that firstly, the most commonly used drugs in the ancestral past are the same drugs that are still most commonly used today, e.g. tobacco, coffee, tea, cannabis, betel nut, khat, coca and cola nut (Sullivan & Hagen 2002), and secondly, that the concentrations of alkaloids like nicotine in the wild (or partially domesticated) species encountered by our ancestors is generally higher than in the domesticated species currently used in the manufacture of commercial brands (Watson 1983).

The issues surrounding domestication and potency are complex and contradictory: domestication of edible plants has employed detoxifying cultural technologies to make food safer and more palatable (Johns 1990), whereas plants used as drugs appear to have been artificially selected for potency, rather than detoxification (Sullivan & Hagen 2002). Although issues of domestication require further research, there is little evidence that the transitions to horticulture and agriculture provided an evolutionary ‘window’ free from human exposure to plant neurotoxins.

### (d) Routes of delivery

It has been argued that the mismatch might not be in the nature of the drugs, but in their method of delivery, e.g. via hypodermic needle to bypass first-pass metabolism (Nesse 2002). However, many drug-containing plants are simply chewed or smoked, and pre-industrial and prehistoric societies often used drug delivery methods, such as free-basing techniques in combination with the buccal–oral route that ensured that these drugs were pure (i.e. chemically unbound) and bypassed first-pass metabolism (Sullivan & Hagen 2002). Given the simplicity of these methods, it is entirely plausible that they have been used as long as there have been cognitively modern people, that is, for more than 100 000 years.

### (e) Animal data of drug reward

Some of the strongest data supporting drug reinforcement models have emerged from decades of research using laboratory animals (Liebman & Cooper 1989). Taking these results at face value, however, does not invalidate our critique. The xenobiotic-metabolizing CYP1, CYP2 and CYP3 families are well represented in all mammals for which data are available, including mice, rats, rabbits and primates, and the evidence for coevolution of neurotoxins and mammalian CYP shows that exposure to high concentrations of plant alkaloids is no more an evolutionary novelty for rats or rabbits than it is for humans (Rat Genome Sequencing Project Consortium 2004). Hence, drug reward is as much as a paradox for laboratory animals as it is for humans.

This insight suggests that we should pay particular attention to animal learning research that has incorporated *evolutionarily* and *ecologically plausible* experimental conditions. For example, the classic studies by Petrinovich & Bolles (1954) and Garcia & Ervin (1968) showed that murine experimental outcomes that contradict conventional classical and instrumental conditioning theory are explicable only when interpreted from an evolutionary perspective. Garcia & Ervin (1968) showed that rats will avoid novel foods paired with an aversive association after a single trial, but only if the aversive experience is nausea. Petrinovich & Bolles (1954) demonstrated that rats find it easier to learn relationships that are consistent with their natural ecology, and will make such associations independently of experimentally induced motivational states such as hunger and thirst. Garcia & Ervin's insights may be particularly relevant to our argument in that plant toxins may have provided the phylogenetic basis for the conditioned taste aversions observed in rats. A third example is the work of Green *et al*. (2002) showing that rats in ‘enriched’ environments (i.e. those that are relatively less artificial) are less inclined to lever press for drug rewards. The common element in all of these studies, experimental conditions that are ecologically ‘normal’ to the laboratory animal, is missing from other studies that have been hugely influential on the theory of drug reward such as Olds & Milner's classic research of electrical brain stimulation in the rat (Olds & Milner 1954).

## 6. Potential resolutions of the paradox: novel directions for future research

Evolutionary rationales that can accommodate current drug-reward models might include that the most commonly used plant drugs today have exploited human pleasurable ‘tastes’ to encourage domestication, much in the way that sweet tasting fruits and nectar promote seed and pollen dispersal by animals (Nesse 2002). Tobacco stands as a counter-example to this hypothosis. Nicotine evolved before humans, is toxic for tobacco herbivores, is induced in tobacco plants when subject to herbivory, and has no known function for seed dispersal by non-human species. Thus, it is unlikely that it evolved to exploit pleasurable tastes of humans or other animals.

Even if psychoactive plant toxins are not an evolutionary novelty, it is possible that a few toxins incidentally activate reward circuitry (Nesse 2002). As discussed previously, however, responses to first use of tobacco and other recreational drugs are usually aversive with nausea and vomiting as a commonly occurring outcome (Eissenberg & Balster 2000; Sullivan & Hagen 2002). In neurobiological terms, most drugs *are* recognized as toxins, suggesting that the paradox of drug reward might be usefully informed by a greater understanding of why aversion mechanisms and aversive learning do not overcome drug reward and reinforcement (Hagen *et al.* in preparation).

Not all neurobiological theories of drug use invoke evolved neural signalling pathways. Theories of chronic drug use emphasize neuroplastic ‘adaptations’ to chronic drug exposure and addiction, and focus less on hedonic feedback circuits that motivate initial drug seeking and responses to acute drug exposure (Kalivas 2005; Shaham & Hope 2005). Such models, as non-evolutionary explanations for habituated drug use, do not necessarily conflict with the evidence of plant–animal coevolution.

### (a) Questioning a unitary reward model

There is a near consensus that all recreational drugs increase dopaminergic transmission in the NAc, however, when compared with opiates, the mechanisms by which they do so are ‘more conjectural’ (Nestler 2005). For instance, mesolimbic and neostriatal dopamine projections are crucial to sensorimotor function, which, in turn, means that sensorimotor responses to dopamine manipulation complicate a clear understanding of dopamine's role in reward (Berridge & Robinson 1998). Furthermore, on the basis of observations that do not easily fit prevailing reward and reinforcement models of the MDS, several researchers have suggested that the MDS involves broader functions such as attention, complex sensorimotor integration, effort or behavioural programme switching (see, e.g. Horvitz 2000; Salamone *et al*. 2005). If so, drug use could be explained by effects other than, or in addition to, reward and reinforcement. A unitary reward model for all drugs of abuse, in other words, is not yet established.

Although the unitary account of drug use provided by the MDS model is elegant, we believe that it would also be beneficial to explore drug-specific mechanisms. It is commonly recognized that different drugs have profoundly different and wide-ranging effects on the CNS and PNS, and it is likely that the explanation for opiate use could differ in fundamental ways from the explanation for tobacco use. For example, it is relatively easier to induce the self-administration of opiates than nicotine in laboratory animals (Le Foll & Goldberg 2006). It is important to keep in mind that the opium poppy evolved morphine to replace endogenous endorphins at *μ*-opioid receptors, and that the tobacco plant evolved nicotine to replace endogenous acetylcholine at cholinergic receptors, in the CNS and PNS (table 1), not to activate the MDS ‘downstream’. If we were to consider each toxin/receptor relationship as a *distinct* ecological phenomenon with, firstly, its own neurochemistry reflecting plant-defence strategies and, secondly, potential consumer counter-strategies, we may find that multiple drug pathway models can account for neurobiological and behavioural data of acute drug states better than the current comprehensive models.

### (b) Ecological approaches to drug use

Thus far, we can summarize certain common elements that should be present in ecological and/or evolutionary approaches to research of substance use as follows.

- Making a distinction between the causes of neophyte use and the physiological and behavioural processes associated with chronic drug use.
- Using information about natural patterns of seeking and use. For example, what are the ecologically salient factors affecting initial use patterns in humans and animals?
- Using independent variables that are ecologically meaningful, or plausible, to the experimental subject; that is, which reflect a plausible aspect of the animal's natural ecology and evolutionary history. For example, the key elements of Garcia & Ervin's (1968) classic study—food paired with nausea—are ‘ecologically plausible’ to the rat; by contrast, the electrical brain stimulation used in Olds & Milner's famous study (1954) has no natural parallel in the ecology or phylogeny of the rat.
- Focusing on the primary neurobiological target of the neurotoxin rather than downstream interactions with dopamine and/or the NAc. For example, the neurotoxin nicotine has evolved to bind with cholinergic receptors, not dopaminergic receptors; what are the primary physiological (and behavioural) correlates of cholinergic receptor binding, and how do they relate to 1, 2 and 3 above?
- Considering the possibility of coevolutionary processes. Animals that are ecologically exposed to plant neurotoxins often evolve strategies and adaptations to counter-exploit the neurotoxin.

In regard to the latter, the broad patterns of human drug-seeking behaviour (see the macro trends in the US National Survey data; figure 1) may reflect active substance seeking mediated by presently unknown toxin-exploiting mechanisms, similar to those found in numerous other species (Karban & Agrawal 2002), rather than an inherent vulnerability to drugs. For example, Bentz & Barbosa (1990) demonstrated that the food use efficiency of unparasitized tobacco hornworm larvae was significantly reduced by ingestion of nicotine. However, larvae that consumed food containing nicotine were themselves protected from parasitism by the wasp *Cotesia congregata*. Parasitized larvae that consumed a nicotine-laced diet had significantly greater efficiency in conversion of ingested and digested food than parasitized larvae without dietary nicotine, presumably because nicotine was worse for the parasitic wasps than it was for the hornworm larvae (an example of what ecologists call *pharmacophagy*). Note that the benefit of nicotine exposure exceeded the cost only when the hornworm was itself parasitized. This example demonstrates both the cost of toxin consumption to co-adapted species (tobacco hornworm/*Nicotiana*) and how a targeted plant predator may counter-exploit the toxins deployed against it.

In this light, it has been proposed that toxins in fava beans and cassava might be effective against *Plasmodium falciparum* infections in humans (Jackson 1990, 1996), that the ubiquitous use of spices could be an adaptation to exploit plant alkaloids to combat bacterial infections of food (Billing & Sherman 1998), and that the evidence that primates and other animals use the toxic properties of plants to self-medicate, especially against gastrointestinal parasites, may provide an evolutionary basis for human medicinal behaviour (Johns 1990, 1999; Huffman 1997). We have hypothesized elsewhere that hominins may have exploited plant toxins to overcome nutritional and energetic constraints on CNS signalling (Sullivan & Hagen 2002).

Here we note that some recreational drugs attack human pathogens. For example, of the world's three most popular alkaloid drugs—caffeine (coffee), nicotine (tobacco) and arecoline (betel nut)—two, in the form of nicotine sulphate and arecoline hydrobromide, are potent commercial anthelmintics used in animals (Eckert *et al*. 1981; Hammond *et al*. 1997). Dried tobacco leaves, stalks and the whole herb are still employed by farmers in parts of the developing world to treat helminth infections in livestock, and it has been shown that an aqueous extract of nicotine from tobacco leaves is quite effective against helminth infections in cattle and sheep (Msolla *et al*. 1987; Iqbal *et al*. 2006). Like nicotine, modern anthelmintics such as levamisole and tetrahydropyrimidines target nicotinic acetylcholine receptors on somatic muscle cells, inducing spastic paralysis and parasite expulsion (Kohler 2001). Nicotinic receptors are also targeted by recently developed ‘neonicotinoid’ agents effective against fleas, ticks and other arthropod parasites (Tomizawa & Casida 2005).

Although we are not aware of any quantitative studies, orally ingested nicotine and arecoline are seen as efficacious anthelmintics in humans (Fabricant & Farnsworth 2001). Thus, the widespread recreational use of plants producing nicotine and arecoline could be an evolved response to chronic infections of parasites (with nicotinic or muscarinic receptors) in ancestral human populations. However, we doubt that selection occurred for use of nicotine or arecoline specifically; it appears more likely that there could have been selection to seek out and use cholinergic agents of various types.^3^ According to this hypothesis, any pleasure or satisfaction from nicotine and arecoline use stems not from incidental activation of a general reward mechanism, but rather from an evolved propensity for ‘human pharmacophagy’ using these or related cholinergic compounds. As in the case of pharmacophagy by the parasitized tobacco hornworm, our ancestors may have initially exploited plant neurotoxins because, although they are ‘bad’ (toxic) for humans, they are even worse for some human pathogens.

Currently, any potential benefits from the antihelmentic properties of nicotine are outweighed by the considerable health costs of tobacco consumption in long-lived, resource-rich Western populations with low parasite loads. In the shorter-lived, nutritionally stressed populations with higher parasite loads, such as those in which our ancestors evolved, the antihelmentic properties of cholinergic plant toxins may have constituted a significant adaptive opportunity. These hypotheses are testable using both descriptive and experimental methods in animals and humans. Animal research could observe a propensity for consuming cholinergic substances after experimental infection with helminths. In humans, observational research could describe associations between consumption of cholinergic substances and rates of helminth infections in natural populations. Experimental studies could measure the effects of cholinergic interventions on experimentally induced (benign) helminth infections or, conversely, the effects of manipulations of existing infection (e.g. by treatment with commercial anthelmintics) on existing cholinergic substance use (e.g. tobacco use).

## 7. Conclusion

This paper has sought to illuminate the paradox between evolutionary biology's punishment model and neurobiology's reward model. Existing models of drug reward have effectively bypassed the paradox by ignoring the evolved function of plant drugs and the probable coevolution of plant defensive compounds and herbivore nervous systems. We have critiqued major assumptions underlying the current evolutionary justification of reward models, that: drugs are an evolutionary novelty; humans (and mammals) are inherently vulnerable; and hedonic reward best characterizes the psychological and physiological responses to drug exposure. Our review has identified several elements of research design that we believe would constitute evolutionarily/ecologically plausible research of substance use, including considering the possibility of counter-exploitation of plant toxins in human evolution. We have also provided an initial hypothesis that human substance seeking may have evolved to exploit the anti-parasitic properties of commonly used plant toxins, but there are, of course, other possibilities.

In our opinion, resolving the paradox of human drug use will require new neurobiological models, or new interpretations of neurobiological reward theory, that are consistent with insights from evolutionary ecology. More generally, we urge neurobiologists studying drug use, and evolutionary biologists studying plant–herbivore interactions, to expand their research agenda to incorporate findings and insights from one another.

## Figures and Tables

**Table 1:** Relationships between CNS receptors and plant neurotoxins commonly used as drugs.

Toxin (typical source)	Receptor
Nicotine^a^ (tobacco)	Nicotinic acetylcholine
Arecoline^a^ (betel nut)	Muscarinic acetylcholine
Cocaine^c^ (coca)	Adrenergic, Dopaminergic
Ephedrine^c^ (khat)	Adrenergic, Dopaminergic
Caffeine^b^ (coffee)	Adenosine
Theophylline^b^ (tea)	Adenosine
Theobromine^b^ (chocolate)	Adenosine
Morphine^a^ (opium poppy)	Opioid
Δ9-THC^a^ (cannabis)	Cannabinoid

a receptor agonist.

b receptor antagonist.

c reuptake inhibitor.

**Table 2:** Examples of human cytochrome P450 enzymes that play an important role in plant drug metabolism. Drugs/toxins are often metabolized by multiple enzymes.

Enzyme / Plant neurotoxin substrate (typical source)
*CYP1A2*
Caffeine (*Coffea*–coffee)
Theophylline (*Camellia sinensis*–tea)
Theobromine (*Theobromine cacao*–chocolate)
*CYP2A6*
Nicotine (*Nicotiana*–tobacco)
Coumarin (*Dipteryx odorata*–tonka bean)
Cotinine (nicotine metabolite)
*CYP2B6*
Nicotine (induces 2B6 in the brain)
Diazepam (synthetic drug; trace amounts in plants)
*CYP2C8*
Taxol (*Taxus brevifolia*)
*CYP2C9*
Δ9-THC (*Cannabis sativa*–cannabis)
*CYP2D6*
Codeine (*Papaver somniferum*–opium poppy)
Harmaline (*Peganum harmala*)
Harmine (*Peganum harmala*)
Sparteine (*Lupinus*)
Yohimbine (*Pausinystalia yohimbe*)
*CYP2E1*
Theobromine (*Theobromine cacao*–chocolate)
*CYP3A4*
Cocaine (*Erythroxylum coca*)
Quinine (*Cinchona*)

**Table 3:** Example ethnic population frequencies of CYP2A6 and CYP2D6 alleles with known *in vivo* enzyme activity. Frequencies compiled from different studies in the same ethnic population are only approximately comparable. Data from Aklillu *et al.* 1996, Gyamfi *et al.* 2005, Haberl *et al.* 2005, Ingelman-Sundberg 2005, Nakajima *et al.* 2002, Yoshida *et al.* 2003.

Allele	Enzyme activity	Population frequencies (%)		
		Caucasian	Japanese	
CYP2A6^*^1A/B	Normal	88.4	48.3	
CYP2A6^*^2	None	2.3	0	
CYP2A6^*^4	None	1.2	20.1	
CYP2A6^*^5	None	0	0	
CYP2A6^*^7	Reduced	0	6.5	
CYP2A6^*^9	Reduced	5.2	21.3	
CYP2A6^*^10	Reduced	0	1.1	
CYP2A6^*^12	Reduced	3	0	
		Caucasian	Asian	Ethiopian
CYP2D6*2xn	Increased	1-5	0-2	16.0
CYP2D6*4	None	12-21	1	1.2
CYP2D6*5	None	2-7	6	3.3
CYP2D6*10	Reduced	1-2	51	8.6
CYP2D6*17	Reduced	0	0	9.0

## Supplementary Material

Additional material Table 1 *Relationships between plant neurotoxins commonly used as drugs and CNS receptors*. Table 2 *Human cytochrome P450 natural substrates and enzyme kinetics constants*. Table 3 *Example ethnic population frequencies of CYP2A6 and CYP2D6 alleles with known in vivo enzyme activity*. References

## Endnotes

1 Our arguments apply equally to evolved fungal defensive compounds (e.g. psilocybin). Ethanol is the only commonly used drug that is not a secondary compound and it therefore falls outside the boundaries of our discussion. See Dudley (2002) for an evolutionary account of ethanol use.

2 The dynamic of co-evolutionary antagonism has several important exceptions such as seed and pollen dispersal, and there is unlikely to be much co-evolution between decomposers and degradable plant detritus.

3 There are a number of cholinergic plant toxins (Wink & Schimmer 1999).
