# Supplementary material for: Revealing the paradox of drug reward in human evolution
Source: Proc Biol Sci. 2008 Mar 19;275(1640):1231–41. doi: 10.1098/rspb.2007.1673 (PMC2367444; doi:10.1098/rspb.2007.1673)
Supplement: Supplementary file 1 [file rspb20071673s01.pdf]

Table 1
Relationships between plant neurotoxins commonly used as drugs and CNS receptors.

Drug	Plant	Toxin	Neurotransmitter	Receptor
Tobacco, Pituri	*Nicotiana, Duboisia*	Nicotine[a]	Acetylcholine	Nicotinic receptor
Betel nut	*Areca catechu*	Arecoline[a]	Acetylcholine	Muscarinic receptor
Coca	*Erythroxylum*	Cocaine[c]	Norepinephrine, epinephrine	Adrenergic receptors
Khat	*Catha edulis*	Ephedrine[c], cathinone[a,c]	Norepinephrine, epinephrine	Adrenergic receptors
Coca	*Erythroxylum*	Cocaine[c]	Dopamine	Dopamine receptor
Khat	*Catha edulis*	Cathinone[a,c]	Dopamine	Dopamine receptor
Coffee, Cola nut	*Coffea, Cola nitida*	Caffeine[b]	Adenosine	Adenosine receptor
Tea	*Camellia sinensis*	Caffeine[b], theophylline[b], theobromine[b]	Adenosine	Adenosine receptor
Chocolate	*Theobromine cacao*	Theobromine[b]	Adenosine	Adenosine receptor
Opium	*Papaver somniferum*	Codeine[a], morphine[a]	Endorphins	Opioid receptor
Cannabis	*Cannabis sativa*	Δ9-THC[a]	Anandamide	Cannabinoid receptor

[a]receptor agonist, [b]receptor antagonist, [c]reuptake inhibitor

Table 2

Human cytochrome P450 natural substrates and enzyme kinetics constants. V_{max} is the maximum reaction rate per unit enzyme. K_m, the Michaelis-Menten constant, is the substrate concentration at which the reaction rate = $V_{max}/2$ (lower values indicate higher enzyme affinity for substrate). V_{max} / K_m is an index of enzyme activity (higher values indicate higher enzyme activity). Kinetic values can vary widely; values here are representative of one metabolic pathway (substrates are typically metabolized via multiple pathways). Plants listed are often not the exclusive source of the neurotoxin.

Enzyme/substrates	Xenobiotic and endogenous sources	$K_m (\mu M)$	V_{max}	V_{max} / K_m
**CYP1A2**				
Caffeine	Plant neurotoxin (*Coffea* – coffee)	460	570^b	1.24
Theophylline	Plant neurotoxin (*Camellia sinensis* – tea)	310	43.3^b	0.14
Theobromine	Plant neurotoxin (*Theobromine cacao* – chocolate)	2580	1720^b	0.67
Aflatoxin B1	Fungus neurotoxin	36	0.92^a	0.026
PhIP	Cooked meat	46	1.79^a	0.039
Estradiol	Sex hormone	27.5	17.4^a	0.63
Melatonin	Hormone	25.9	10.6^a	0.41
**CYP2A6**				
Nicotine	Plant neurotoxin (*Nicotiana* – tobacco)	95.3	154.1^b	1.69
Coumarin	Plant neurotoxin (*Dipteryx odorata* – tonka bean)	0.6	0.6^a	1.0
Cotinine	Nicotine metabolite	234.5	37.2^b	0.16
**CYP2B6**				
Nicotine	Plant neurotoxin [induces 2B6 in the brain]			
Diazepam	Synthetic drug; trace amounts in plants	181	8.5^a	0.05
**CYP2C8**				
Taxol	Plant neurotoxin (*Taxus brevifolia*)	5.4	30^a	5.6
Arachidonic acid	Essential omega-6 fatty acid	71	0.078^a	0.001
Retinol	Vitamin A	50	1.2^a	0.024
**CYP2C9**				
Δ9-THC	Plant neurotoxin (*Cannabis sativa* – marijuana)	2.1	6.4^a	3.0
**CYP2C19**				
Melatonin	Hormone	282.2		
Progesterone	Hormone	3.6	1.4^a	0.39
**CYP2D6**				
Codeine	Plant neurotoxin (*Papaver somniferum* – opium poppy)	190	6.4^a	0.034
Harmaline	Plant neurotoxin (*Peganum harmala*)	1.41	39.9^a	28.3
Harmine	Plant neurotoxin (*Peganum harmala*)	7.42	29.7^a	4.0
Sparteine	Plant neurotoxin (*Lupinus*)	44		
Yohimbine	Plant neurotoxin (*Pausinystalia yohimbe*)	2.0	147.4^b	75.5
**CYP2E1**				
Theobromine	Plant neurotoxin (*Theobromine cacao* – chocolate)	3400		
Ethanol	Yeast waste product	23400	23.2^a	0.001
**CYP3A4**				
Cocaine	Plant neurotoxin (*Erythroxylum coca*)	2700	3744.4^b	1.4
Quinine	Plant neurotoxin (*Cinchona*)	106	1330^b	13
Aflatoxin B1	Fungus neurotoxin	139	61^a	0.45
Testosterone	Hormone	52	5400^b	101
Cortisol	Hormone	15	6.4^b	0.42

^{a} *pmol/min/pmol* P450; ^{b} *pmol/min/mg* microsomal protein

Data from Bland, Haining, Tracy, and Callery (2005); Bu (2006); Gates and Miners (1999); Ladona, Gonzalez, Rane, Peter, and Torre (2000); Le Corre et al. (2004); Lewis (2001, 2003); Osikowska-Evers and Eichelbaum (1986); Projean, Morin, Tu, and Ducharme (2003); Yang et al. (1998); Yu, Kneller, Rettie, and Haining (2002); Yu, Idle, Krausz, Kupfer, and Gonzalez (2003); Ma, Idle, Krausz, and Gonzalez (2005); Usmani, Cho, Rose, and Hodgson (2006); Yamazaki and Shimada (1997); Bloomer, Clarke, and Chenery (1995); Murphy, Raulinaitis, and Brown (2005); Hammons et al. (1997); Nakajima et al. (1996, 1996); Tjia, Colbert, and Back (1996); Ha, Follath, Chen, and Krähenbühl (1996); Asai, Imaoka, Kuroki, Monna, and Funae (1996); Marill, Cresteil, Lanotte, and Chabot (2000); Rahman, Korzekwa, Grogan, Gonzalez, and Harris (1994); Campbell, Grant, Inaba, and Kalow (1987); Gallagher, Kunze, Stapleton, and Eaton (1996).

Table 3
Example ethnic population frequencies of CYP2A6 and CYP2D6 alleles with known in vivo *enzyme activity. Frequencies compiled from different studies in the same ethnic population are only approximately comparable.*

Allele	Enzyme activity	Population frequencies (%)				
		Ghanaian[1]	Caucasian[2,3]	Chinese[1,2,3]	Japanese[1,2,3,4]	
CYP2A6*1A/B	Normal	91.9	88.4	61.7	48.3	
CYP2A6*2	None	0	2.3	0	0	
CYP2A6*4	None	1.9	1.2	15.1	20.1	
CYP2A6*5	None	0	0	1	0	
CYP2A6*7	Reduced	0	0	2.2	6.5	
CYP2A6*9	Reduced	5.7	5.2	15.7	21.3	
CYP2A6*10	Reduced	0	0	0.4	1.1	
CYP2A6*12	Reduced		3	0	0	
		Black African[5]	Caucasian[5]	Asian[5]	Ethiopian[6]	Saudi Arabian[7]
CYP2D6*2xn	Increased	2	1-5	0-2	16.0	10.4
CYP2D6*4	None	2	12-21	1	1.2	3.5
CYP2D6*5	None	4	2-7	6	3.3	1.0
CYP2D6*10	Reduced	6	1-2	51	8.6	3.0
CYP2D6*17	Reduced*	20-35	0	0	9.0	3.0

1: Gyamfi, Fujieda, Kiyotani, Yamazaki, and Kamataki (2005); 2: Nakajima, Kuroiwa, and Yokoi (2002);
3: Haberl et al. (2005); 4: Yoshida et al. (2003); 5: Ingelman-Sundberg (2005); 6: Aklillu et al. (1996);
7: McLellan, Oscarson, Seidegård, Evans, and Ingelman-Sundberg (1997)

References

Aklillu, E., Persson, I., Bertilsson, L., Johansson, I., Rodrigues, F., and Ingelman-Sundberg, M. (1996). Frequent distribution of ultrarapid metabolizers of debrisoquine in an Ethiopian population carrying duplicated and multiduplicated functional CYP2D6 alleles. *J Pharmacol Exp Ther*, 278:441–446.

Asai, H., Imaoka, S., Kuroki, T., Monna, T., and Funae, Y. (1996). Microsomal ethanol oxidizing system activity by human hepatic cytochrome P450s. *J Pharmacol Exp Ther*, 277(2):1004–1009.

Bland, T., Haining, R., Tracy, T., and Callery, P. (2005). CYP2C-catalyzed delta9-tetrahydrocannabinol metabolism: kinetics, pharmacogenetics and interaction with phenytoin. *Biochem Pharmacol*, 70(7):1096–103.

Bloomer, J. C., Clarke, S. E., and Chenery, R. J. (1995). Determination of P4501A2 activity in human liver microsomes using [3-14C-methyl] caffeine. *Xenobiotica*, 25(9):917–27.

Bu, H. Z. (2006). A literature review of enzyme kinetic parameters for CYP3A4-mediated metabolic reactions of 113 drugs in human liver microsomes: Structure-kinetics relationship assessment. *Current Drug Metabolism*, 7(3):231–249.

Campbell, M. E., Grant, D. M., Inaba, T., and Kalow, W. (1987). Biotransformation of caffeine, paraxanthine, theophylline, and theobromine by polycyclic aromatic hydrocarbon-inducible cytochrome (s) P-450 in human liver microsomes. *Drug Metabolism and Disposition*, 15(2):237–249.

Gates, S. and Miners, J. O. (1999). Cytochrome P450 isoform selectivity in human hepatic theobromine metabolism. *British Journal of Clinical Pharmacology*, 47(3):299–305.

Gyamfi, M., Fujieda, M., Kiyotani, K., Yamazaki, H., and Kamataki, T. (2005). High prevalence of cytochrome P 450 2A6* 1A alleles in a black African population of Ghana. *European Journal of Clinical Pharmacology*, 60(12):855–857.

Ha, H. R., Follath, F., Chen, J., and Krähenbühl, S. (1996). Biotransformation of caffeine by cDNA-expressed human cytochromes P-450. *European Journal of Clinical Pharmacology*, 49(4):309–315.

Haberl, M., Anwald, B., Klein, K., Weil, R., Fuss, C., Gepdiremen, A., Zanger, U., Meyer, U., and Wojnowski, L. (2005). Three haplotypes associated with CYP2A6 phenotypes in Caucasians. *Pharmacogenet Genomics*, 15:609–624.

Hammons, G. J., Milton, D., Stepps, K., Guengerich, F. P., Tukey, R. H.,

and Kadlubar, F. F. (1997). Metabolism of carcinogenic heterocyclic and aromatic amines by recombinant human cytochrome P450 enzymes. *Carcinogenesis*, 18:851–854.

Ingelman-Sundberg, M. (2005). Genetic polymorphisms of cytochrome P450 2D6 (CYP2D6): clinical consequences, evolutionary aspects and functional diversity. *The Pharmacogenomics Journal*, 5:6–13.

Ladona, M., Gonzalez, M., Rane, A., Peter, R., and de la Torre, R. (2000). Cocaine metabolism in human fetal and adult liver microsomes is related to cytochrome P450 3A expression. *Life Sci*, 68(4):431–43.

Le Corre, P., Parmer, R., Kailasam, M., Kennedy, B., Skaar, T., Ho, H., Leverge, R., Smith, D., Ziegler, M., Insel, P., et al. (2004). Human sympathetic activation by 〈2-adrenergic blockade with yohimbine: Bimodal, epistatic influence of cytochrome P450–mediated drug metabolism. *Clinical Pharmacology & Therapeutics*, 76(2):139–153.

Lewis, D. F. V. (2001). *Guide to Cytochromes P450: Structure and Function*. Taylor and Francis, London.

Lewis, D. F. V. (2003). Human cytochromes P450 associated with the phase 1 metabolism of drugs and other xenobiotics: A compilation of substrates and inhibitors of the CYP1, CYP2 and CYP3 families. *Current Medicinal Chemistry*, 10:1955–1972.

Ma, X., Idle, J. R., Krausz, K. W., and Gonzalez, F. J. (2005). Metabolism of melatonin by human cytochromes P450. *Drug Metab Dispos*, 33(4):489–94.

Marill, J., Cresteil, T., Lanotte, M., and Chabot, G. G. (2000). Identification of human cytochrome P450s involved in the formation of all-trans-retinoic acid principal metabolites. *Molecular Pharmacology*, 58(6):1341.

McLellan, R. A., Oscarson, M., Seidegård, J., Evans, D. A., and Ingelman-Sundberg, M. (1997). Frequent occurrence of CYP2D6 gene duplication in Saudi Arabians. *Pharmacogenetics*, 7:187–91.

Murphy, S., Raulinaitis, V., and Brown, K. (2005). Nicotine 5’-oxidation and methyl oxidation by P450 2A enzymes. *Drug Metab Dispos*, 33(8):1166–1173.

Nakajima, M., Kuroiwa, Y., and Yokoi, T. (2002). Interindividual differences in nicotine metabolism and genetic polymorphisms of human CYP2A6. *Drug Metabolism Reviews*, 34(4):865–877.

Nakajima, M., Yamamoto, T., Nunoya, K., Yokoi, T., Nagashima, K., Inoue, K., Funae, Y., Shimada, N., Kamataki, T., and Kuroiwa, Y. (1996a). Characterization of CYP2A6 involved in 3’-hydroxylation

of cotinine in human liver microsomes. *J Pharmacol Exp Ther*, 277(2):1010–1015.

Nakajima, M., Yamamoto, T., Nunoya, K., Yokoi, T., Nagashima, K., Inoue, K., Funae, Y., Shimada, N., Kamataki, T., and Kuroiwa, Y. (1996b). Role of human cytochrome P4502A6 in C-oxidation of nicotine. *Drug Metab Dispos*, 24(11):1212–1217.

Osikowska-Evers, B. and Eichelbaum, M. (1986). A sensitive capillary GC assay for the determination of sparteine oxidation products in microsomal fractions of human liver. *Life Sci*, 38(19):1775–82.

Projean, D., Morin, P. E., Tu, T. M., and Ducharme, J. (2003). Identification of CYP3A4 and CYP2C8 as the major cytochrome P450s responsible for morphine N-demethylation in human liver microsomes. *Xenobiotica*, 33(8):841–854.

Rahman, A., Korzekwa, K. R., Grogan, J., Gonzalez, F. J., and Harris, J. W. (1994). Selective biotransformation of taxol to 6 alpha-hydroxytaxol by human cytochrome P450 2C8. *Cancer Research*, 54(21):5543–5546.

Tjia, J. F., Colbert, J., and Back, D. J. (1996). Theophylline metabolism in human liver microsomes: inhibition studies. *J Pharmacol Exp Ther*, 276(3):912–917.

Usmani, K. A., Cho, T. M., Rose, R. L., and Hodgson, E. (2006). Inhibition of the human liver microsomal and human cytochrome P450 1A2 and 3A4 metabolism of estradiol by deployment-related and other chemicals. *Drug Metabolism and Disposition*, 34(9):1606–1614.

Yamazaki, H. and Shimada, T. (1997). Progesterone and testosterone hydroxylation by cytochromes P450 2C19, 2C9, and 3A4 in human liver microsomes. *Archives of Biochemistry and Biophysics*, 346(1):161–169.

Yang, T. J., Shou, M., Korzekwa, K. R., Gonzalez, F. J., Gelboin, H. V., and Yang, S. K. (1998). Role of cDNA-expressed human cytochromes P450 in the metabolism of diazepam. *Biochemical Pharmacology*, 55(6):889–896.

Yoshida, R., Nakajima, M., Nishimura, K., Tokudome, S., Kwon, J.-T., and Yokoi, T. (2003). Effects of polymorphism in promoter region of human CYP2A6 gene (CYP2A6*9) on expression level of messenger ribonucleic acid and enzymatic activity in vivo and in vitro. *Clinical Pharmacology and Therapeutics*, 74:69–76.

Yu, A., Idle, J., Krausz, K., Kupfer, A., and Gonzalez, F. (2003). Contribution of individual cytochrome P450 isozymes to the O-demethylation of the psychotropic ®-carboline alkaloids

harmaline and harmine. *Journal of Pharmacology and Experimental Therapeutics*, 305(1):315–322.

Yu, A., Kneller, B., Rettie, A., and Haining, R. (2002). Expression, purification, biochemical characterization, and comparative function of human cytochrome P450 2D6. 1, 2D6. 2, 2D6. 10, and 2D6. 17 allelic isoforms. *Journal of Pharmacology and Experimental Therapeutics*, 303(3):1291–1300.
